# An Analytical Model for the Aggregate Throughput of IEEE 802.11ah Networks under the Restricted Access Window Mechanism †

**DOI:** 10.3390/s22155561

**Published:** 2022-07-26

**Authors:** Stephanie M. Soares, Marcelo M. Carvalho

**Affiliations:** Department of Electrical Engineering, University of Brasilia, Brasilia 70297-400, Brazil; mmcarvalho@ene.unb.br

**Keywords:** IEEE 802.11ah, Internet of Things, wireless networks, analytical modeling

## Abstract

The IEEE 802.11ah is an amendment to the IEEE 802.11 standard to support the growth of the Internet of Things (IoT). One of its main novelties is the restricted access window (RAW), which is a channel access feature designed to reduce channel contention by dividing stations into RAW groups. Each RAW group is further divided into RAW slots, and stations only attempt channel access during the RAW slot they were assigned to. In this paper, we propose a discrete-time Markov chain model to evaluate the average aggregate throughput of IEEE 802.11ah networks using the RAW mechanism under saturated traffic and ideal channel conditions. The proposed analytical model describes the behavior of an active station within its assigned RAW slot. A key aspect of the model is the consideration of the event of RAW slot time completion during a station’s backoff operation. We study the average aggregate network throughput for various numbers of RAW slots and stations in the network. The numerical results derived from our analytical model are compared to computer simulations based on an IEEE 802.11ah model developed for the ns-3 simulator by other researchers, and its performance is also compared to two other analytical models proposed in the literature. The presented results indicate that the proposed analytical model reaches the closest agreement with independently-derived computer simulations.

## 1. Introduction

Currently, wireless networks are becoming increasingly pervasive, extending their reach to uncharted territories such as farms, manufacturing plants, retail stores, warehouses, and autonomous vehicles, to name a few, aiming to provide access to a multitude of objects and unleash the full potential of the Internet of Things (IoT) [1,2,3,4,5,6,7]. To create a smart environment, IoT technology has led to a growing interconnection between devices at a scale and speed never seen before [8,9]. This unprecedented number of over-the-air connections poses significant challenges to the design of next-generation wireless networks due to the need to support (i) a massive amount of concurrent connections and data traffic, (ii) highly heterogeneous and stringent quality-of-service (QoS) requirements, and (iii) efficient and fair use of scarce network resources (e.g., energy and bandwidth) [10]. In this effort, it is envisaged that Wireless Local Area Networks (WLANs) will play an important role in the deployment and dissemination of IoT applications because Wi-Fi devices have been widely adopted and can operate in the unlicensed spectrum [11]. However, current Wi-Fi networks (e.g., IEEE 802.11ac/ax) face many challenges in IoT scenarios, which generally consist of large-scale networks deployed over areas that are wider than traditional WLANs [12]. Moreover, as the number of competing stations increases, traditional IEEE 802.11 networks become less efficient due to a higher probability of data packet collisions, leading to significant performance degradation. Motivated by such IoT features, the IEEE 802.11ah amendment [13] was developed to operate on sub-GHz frequency channels in order to support higher network coverage. In addition, it introduces a number of other features that allow up to 8191 stations to be associated with a single access point under higher energy efficiency, which makes it an attractive wireless access technology for IoT applications.

The IEEE 802.11ah standard inherited the physical layer of the IEEE 802.11ac, with adaptations to operate at frequencies below 1 GHz, and channel bandwidths ranging from 1 MHz to 16 MHz, with the 1 MHz and 2 MHz bands being widely adopted. In particular, many new features were incorporated into the MAC sub-layer in order to provide better support for energy consumption and channel access under dense network scenarios, such as the mechanisms of the *restricted access window* (RAW), *traffic indication map* (TIM), and *target wake time* (TWT), along with reduced frame headers and faster association and authentication procedures. In fact, the RAW mechanism is one of the main innovations implemented to handle dense scenarios, by which stations are grouped into “RAW groups,” which are further divided into RAW slots. Only stations assigned to a given RAW slot can compete for channel access during the occurrence of this specific time slot. The assignment of stations to RAW groups and RAW slots, as well as their number and time duration, are all specified by the base station in the so-called *RAW Parameter Set* (RPS) transmitted in the beginning of each beacon interval (whose time duration can also be changed dynamically).

Due to the importance and growing interest in this standard [14,15,16], a number of works have proposed analytical models to evaluate the performance of IEEE 802.11ah networks, especially with respect to the operation of the RAW mechanism. However, in general, previous analytical models have either included features that are not specified in the IEEE 802.11ah standard or have not been validated against some independently-developed simulator that is widely adopted by the networking community. In fact, many previous works have validated their results based on their own customized simulators, whose accuracy and compliance to standard specifications have not been demonstrated. In this sense, the validation of an analytical model against a standard-compliant, well-known simulator is key to establishing its prediction accuracy. Section 2 discusses a number of works related to the analytical modeling of IEEE 802.11ah networks, especially regarding the consideration of the RAW mechanism.

Previously, we have proposed an analytical model for the operation of an IEEE 802.11ah station within its assigned RAW slot under saturated traffic and ideal channel conditions [17]. The proposed model was based on a discrete-time Markov chain formulation that explicitly takes into account the issue of RAW slot time completion, i.e., the moment after which the station can no longer access the channel and thus must wait for its assigned RAW slot in the next beacon interval to contend for channel access again. While the numerical results obtained with the previous analytical model showed close agreement with simulations carried out in the ns-3 simulator [18] and based on an independently-derived IEEE 802.11ah module [19], it still contains some limitations. In particular, its key parameter, the “probability of RAW slot time completion”, did not explicitly consider its dependence on the effective time length of a given RAW slot, and the average throughput expression was not general enough to consider different RAW groups with corresponding RAW slots. Moreover, the performance of the analytical model was not compared against other analytical models proposed in the literature, which is a key step to determining the actual efficacy and strength of our analytical model.

Considering the aforementioned limitations, in this paper, we extend our analytical model by introducing important modifications. First, we improve the analytical model by explicitly considering the impact of the effective time length of a station’s RAW slot in the probability of “RAW slot time completion”. Second, the importance of this probability to the performance of our analytical model is demonstrated in a specific section dedicated to comparisons with computer simulations by considering two cases for our model: when such probability is taken into account, and when it is removed from the model. Third, the analytical development for the computation of the average aggregate throughput is made more general by allowing the consideration of different RAW groups with corresponding RAW slots. Fourth, we compare the average aggregate throughput predicted by our analytical model with results provided by two well-known analytical models available in the literature: Zheng’s model [20] is one of the first works that addresses synchronized grouping of stations, and it is based on a mean value analysis approach, while Sangeetha’s model [21] is a more recent work that follows an approach similar to ours, based on a discrete-time Markov chain model for a station’s backoff operation. We chose these models for comparison because they are fully-specified analytical models that make different assumptions regarding protocol behavior and treat the event of RAW slot time completion differently, which is key for comparison with our model. Incidentally, it is worth mentioning that Ali et al. [22] have proposed an analytical model that contains a probability parameter that plays a similar role to the one we proposed independently [17] for the “RAW slot time completion”. Unfortunately, their work does not provide an explicit expression for the computation of this probability (represented by the parameter $P_{l}$ in the transition probabilities of their Markov Chain in Equations (7), (8), (11) and (12) in [22]), which makes it unsuitable for comparison.

In summary, this paper extends our previous work by providing the following key contributions:

- The analytical model is improved to consider the impact of the effective time length of a station’s RAW slot into the probability of “RAW slot time completion”, which is a key parameter designed to better reflect the station’s dynamics in its assigned RAW slot;
- The key role of the probability of “RAW slot time completion” to our model is demonstrated via comparison with computer simulations by considering the cases when such probability is either included or not in the model;
- We derive a general expression for the computation of the average aggregate throughput over a set of RAW groups and corresponding RAW slots;
- Numerical results of the new analytical model are compared with ns-3 simulations based on the well-known IEEE 802.11ah module independently developed by Le Tian et al. [19,23];
- The prediction accuracy of our new analytical model is shown to be significantly better than the prediction delivered by two other well-known analytical models available in the literature.

The rest of the paper is organized as follows: Section 2 discusses related works, while Section 3 provides an overview of the main features of the IEEE 802.11ah. Section 4 details our analytical model, and in Section 5, we provide a brief description of the two analytical models we use to compare with our own. Section 6 presents the numerical results, while the conclusions of our paper and a discussion of future works are presented in Section 7 and Section 8, respectively.

## 2. Related Work

The analytical modeling of IEEE 802.11ah networks using the RAW mechanism has been a topic of growing interest in the past few years. In the following, we present the main analytical models proposed to date.

Zheng et al. [20] proposed an analytical model based on mean value analysis (MVA) to estimate the average network throughput under saturated traffic conditions. They considered both the cases for the *cross slot boundary* (CSB) mechanism: if enabled, it allows a station to proceed with a packet transmission that can exceed the station’s allocated RAW slot time. Otherwise, a Markov chain model was proposed to estimate the number of mini-slots occupied by the last data transmission in the RAW slot, so that the duration of the next RAW slot can be calculated. They validated their analytical model with simulation results drawn from their own customized simulator.

Qutab-ud-din et al. [24] analyzed the performance of the RAW mechanism when the CSB is disabled and proposed four possible backoff schemes in the holding period to improve the throughput and energy efficiency of saturated IEEE 802.11ah networks. They used Zheng’s [20] analytical model as a benchmark to validate their simulation model developed in the OMNET++ simulator. Therefore, no new analytical model was actually proposed. Mahesh and Harigovindan [25] also used Zheng’s model to propose a grouping scheme based on the average transmission time requirements of devices with the assignment of a priority level to each defined group. Then, they extended Zheng’s analytical model to deal with the average throughput of a single group. They evaluated the average aggregate data transfer by comparing their proposed grouping scheme with uniform grouping using the ns-3 simulator.

Raeesi et al. [26] proposed an analytical model that assumes saturated traffic conditions to estimate the throughput and energy consumption for both basic and four-way handshake mechanisms. They investigated the performance gains obtained with the RAW mechanism compared to the regular operation of the distributed coordination function (DCF). However, their model does not consider the number of nodes as a parameter, and the probability of packet collisions is an input value, not a function of the number of nodes and channel contention. Moreover, the RAW mechanism is not represented in the model. Raeesi et al. [27] also worked on the scenario of multiple IEEE 802.11ah access points with a relatively high number of associated stations. Unfortunately, the proposed analytical model only deals with a theoretical maximum throughput that assumes no packet collisions (i.e., the backoff contention window never increases) and, again, the RAW mechanism is not incorporated in the model.

Park et al. [28] developed an algorithm to estimate the number of devices in the uplink access of IEEE 802.11ah networks in order to determine the optimal duration of the RAW slot. However, they used the original analytical model for the IEEE 802.11 DCF provided by Bianchi [29] to represent the behavior of traffic-saturated stations in the IEEE 802.11ah network. Therefore, the analytical model does not take into account the RAW mechanism explicitly. Moreover, they compared the performance of the uplink total success probability delivered by their algorithm with the legacy (fixed) scheme via analysis and simulations, based on their own simulator.

Sangeetha et al. [21] adopted a discrete-time Markov chain model for the backoff operation of a station in its assigned RAW slot under saturated and non-saturated traffic conditions, using Bianchi’s approach [29] to compute the average throughput. Their model focuses on the case when the CSB is disabled and assumes that the backoff counter stops at the end of the RAW slot to resume its operation in the next assigned RAW slot, starting from the frozen backoff counter value. Thus, when the backoff counter reaches the value 1, the station checks whether the remaining time within the RAW slot is enough to transmit the whole data packet. If not, the station goes to a “defer state” and waits for its next assigned RAW slot in the following beacon interval. However, the described freezing of the backoff counter value is not defined in the standard. Instead, the station is supposed to discard the backoff counter value at the end of a RAW slot and start a new one in the beginning of the next RAW slot. The proposed analytical model was validated based on the ns-3 simulator. Later, they extended their analytical model to address the grouping of stations according to traffic priority [30].

Ali et al. [31] studied the efficiency of the RAW mechanism under non-ideal channel conditions and non-saturated traffic by proposing a Markov chain and an M/G/1 queue-based analytical model. In [22], Ali et al. [22] proposed a discrete-time Markov chain model for IEEE 802.11ah to evaluate the performance of heterogeneous IoT networks with different QoS traffic demands. They considered the case of a disabled CSB and used the same idea as Sangeetha et al. [21] regarding a “defer state” (alternatively named as “delay state”) in case the remaining time within a RAW slot is not enough for data packet transmission. Nevertheless, unlike Sangeetha’s work, the backoff counter is reset in the beginning of every RAW slot. In addition, similar to our previous work [17], they defined a state transition probability to capture the ending of a RAW slot at any given moment during a station’s backoff operation, which makes it return to an initial state for the following RAW slot. Unfortunately, their work did not provide an expression to compute this probability or a technique to estimate it, which makes their work unsuitable for comparison. Also using the idea of a defer state, Bardala and Harigovindan [32] proposed a discrete Markov chain for the backoff operation of a station in a RAW slot. In their work, the station checks for sufficient residual time for transmission at each state of the backoff counter. If the residual time is not sufficient for the transmission, the station goes to a defer state. They also proposed a RAW grouping scheme for a multi-rate network that groups the stations based on their data rate. They used the model to analyze the aggregate throughput of the network for the case where the stations are randomly grouped; then, they compared it with the aggregate throughput obtained with their proposed grouping scheme. They validated their model with ns-3 simulations.

Khorov et al. [33] proposed a discrete-time Markov chain model to find the probability distribution of the time needed for an arbitrary station to successfully transmit its frame when the CSB is disabled. Based on that, they developed the probability distribution for the time needed by all stations to transmit their packets successfully in order to determine the minimum duration of a RAW slot to improve channel efficiency. In [34], they adopted the approach from [33] for the case when the CSB is enabled and and studied the influence of enabling the CSB option. Later, they extended their work to a more general scenario [35], with various traffic patterns and RAW configurations, and analyzed network performance in terms of throughput, energy consumption, and packet loss ratio. For that, they proposed a Markov chain model based on the time slot status, i.e., whether it is occupied by a successful transmission, packet collision, or idle state, and defined absorbing conditions to model the completion time of a RAW slot. They validated their model based on a simulator designed by themselves, i.e., not widely adopted by the networking community. In [36], Bankov et al. extended the model from [35] for the scenario where the IEEE 802.11ah network has energy-harvesting sensor stations. They studied how to optimize the RAW parameters, such as the duration and number of groups, that provide the required probability of data delivery and minimize the amount of consumed channel resources in a network with a high number of energy-harvesting sensor devices.

Nawaz et al. [37] presented a model where a RAW group is divided into two sub-groups and the duration of RAW slots in each sub-group is chosen according to the size of the group to improve network throughput. They showed that overall throughput is improved by assigning a relatively smaller RAW slot duration to a larger size group. They also used Bianchi’s model [29] to compute the network throughput and validated the model with their own simulator. Lakshmi and Sikdar [38] proposed a fair scheduling grouping scheme with different traffic patterns. Their proposal is to assign a weight to the groups according their amount of service, i.e., the amount of data the stations of the group generate per second. In this way, the groups that received higher weights should get more access to the channel than the groups with lower weights. They also used Bianchi’s model [29] to compute the network throughput, and they validated their model using the ns-3 module of Le Tian et al. [19]. Finally, Kai et al. [39] used Bianchi’s approach to formulate throughput and energy efficiency expressions and designed a traffic distribution-based grouping scheme to balance the energy efficiency and fairness guarantees among groups in heterogeneous IEEE 802.11ah networks. They proposed the Optimal Traffic Grouping Algorithm (OTGA) based on an integer nonlinear programming problem and validated their solution with simulations carried out on a simulator designed by themselves.

Station grouping ideas to optimize the protocol performance are also being extensively explored in works related to IEEE 802.11ah. Chang et al. [40] argued that the grouping mechanism performance is related to the different traffic demands of the devices, so the groups must be adapted to the demands of traffic. In this way, they proposed a grouping scheme based on load balancing and verified an improvement in the efficiency in the use of the channel and in throughput. Finally, Dong et al. [41] and Yoon et al. [42] developed grouping schemes according to the geographical location of the devices to avoid collisions related to the hidden terminal problem. Le Tian et al. [43] designed the Traffic-Aware RAW Optimization Algorithm (TAROA), a real-time grouping algorithm for dynamic and heterogeneous traffic. In its original version, TAROA derives its optimal parameters based on saturated state simulations using an improved version of their IEEE 802.11ah ns-3 module [23], and it only supports homogeneous stations under the same modulation and coding scheme (MCS) and average packet size. Their follow-up work [44] allows non-saturated conditions and stations with different MCS and average packet sizes.

## 3. Overview of the IEEE 802.11ah

**Figure 1:**
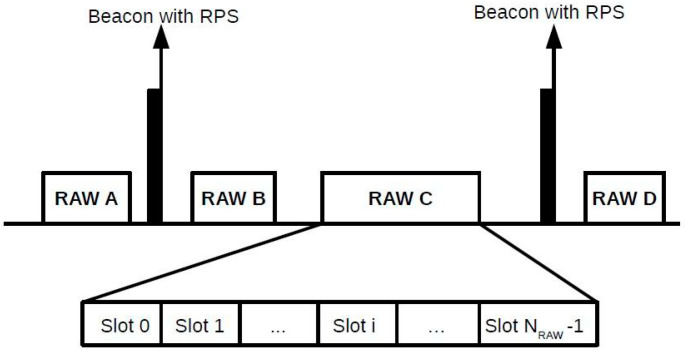
RAW mechanism of IEEE 802.11ah. Each beacon interval is preceded by a RPS beacon. The beacon interval can have one or more RAW groups, and each RAW group can be divided into one or more RAW slots.

The release of the IEEE 802.11ah standard has introduced several new features that aim to facilitate the widespread deployment of IoT applications. As in traditional IEEE 802.11 power save mode, time is divided into beacon intervals, which can have one or more RAW groups, and each RAW group also has its time divided into one or more RAW slots, which are assigned to stations. Only the stations assigned to a given RAW slot can attempt to access the channel during that RAW slot. The beacon intervals can also have periods that are not occupied by RAW groups, in which all stations in the network can attempt channel access. Each beacon interval is preceded by the *RAW Parameter Set* (RPS) beacon that carries RAW information. The RPS specifies characteristics such as which stations belong to the RAW group, the RAW duration, and the number of RAW slots. In addition, the standard defines the *Cross Slot Boundary* (CSB), which indicates if a data packet transmission may exceed the RAW slot duration or not. If the CSB is disabled, a data packet transmission time cannot cross the RAW slot limit. Otherwise, a data packet transmission is permitted to cross the current RAW slot boundary even if it occupies the channel for the next RAW slot period. With the CSB disabled, the RAW slot is divided into two periods: the free access period, in which the stations can attempt to access the channel, and the holding period $T_{h}$, which is a period that is unavailable for stations to contend for channel access because there is not enough time for a packet transmission. The standard also defines a guard period $T_{g}$ between RAW slots to prevent a transmission from one RAW slot to overlap the following RAW slot due to the propagation delay. The RAW mechanism is shown in Figure 1, which shows that a beacon interval can have one or more RAW groups, and each RAW group can be divided into one or more RAW slots.

Unlike the other IEEE 802.11 standards, each station uses two backoff functions according to the Enhanced Distributed Channel Access (EDCA). The first backoff function is used in periods that are not occupied by RAW groups, and all stations in the network are allowed to freely access the channel. The second backoff function is activated inside the RAW slot the station was assigned to. The stations execute the first backoff function during the period of free access to the channel and suspend it in the beginning of the occurrence of a RAW group. When a RAW group period starts, the stations that are not assigned to this RAW group go into the sleep state and save the state at which their first backoff function has stopped, so they can resume it at the end of the time allocated to this RAW group. The stations assigned to the RAW group wake up and start the second backoff function within their assigned RAW slot. When the assigned RAW slot ends, the stations go back to sleep for the rest of the RAW group period. If the RAW slot ends during the execution of the second backoff process, the stations must discard the state in which they were stopped and start a new backoff function in the next assigned RAW slot. Inside its assigned RAW slot, the station selects a random backoff interval according to the binary exponential backoff (BEB) algorithm, which sets it according to

(1) Backoff time=Random()×Slot time,

where Random() generates an integer value uniformly distributed in the interval [0,CW−1], where $CW_{min}\leq CW\leq CW_{max}$, and $CW_{min}$ and $CW_{max}$ are the minimum and maximum contention window sizes, respectively. According to the BEB algorithm, when the channel is idle, the backoff counter is decremented and, if the channel is busy, this counter freezes until the channel is idle again for a time interval greater than or equal to DIFS. Initially, the contention window size is set to the minimum value and, for each unsuccessful transmission attempt, the minimum contention window increases exponentially according to $CW=2^{i}CW_{min}$, where i∈[0,m] and *m* is the maximum number of re-transmission attempts, i.e., $CW_{max}=2^{m}CW_{min}$. Finally, when the backoff counter reaches the value zero, the station can transmit its data packet.

**Figure 2:**
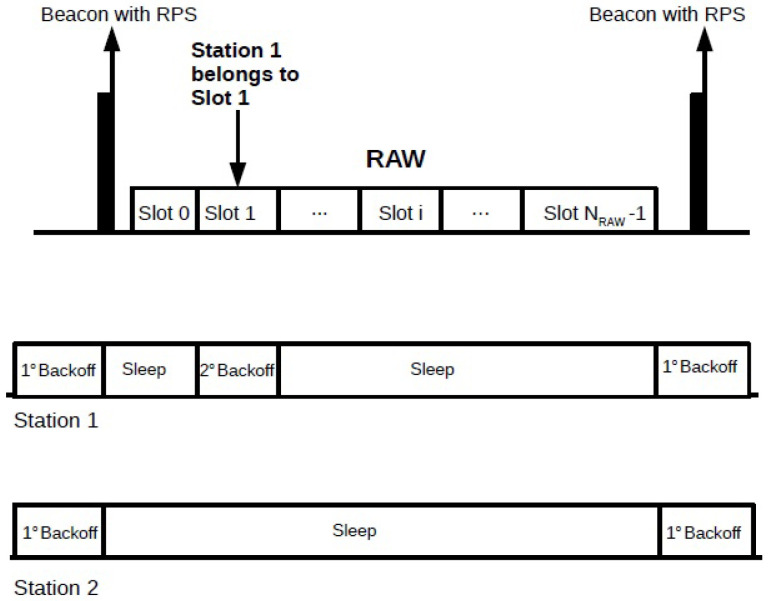
Backoff procedure inside a RAW group. Station 1 belongs to the single RAW group, and it is assigned to RAW slot 1, while station 2 does not belong to this RAW group. Both stations execute the first backoff outside the RAW group period. When the occurrence of the RAW group begins, both stations go into the sleep state. Station 1 wakes up and performs the second backoff within RAW slot 1 and sleeps during the remaining RAW group period. Station 2 remains in sleep state during the entire RAW group period.

Figure 2 shows an example of when the two different backoff functions are activated. Station 1 belongs to the single RAW group shown in the figure, between beacons, where it is assigned to RAW slot 1. Suppose that there is a second station not assigned to this RAW group; then, when the occurrence of the RAW group begins, both stations stop the first backoff counter and go to sleep. During the occurrence of the RAW group period, station 1 wakes up to perform the second backoff within RAW slot 1 and sleeps during the remaining RAW period, while station 2 remains in sleep state during the whole RAW group period because it does not belong to this RAW group. Both stations return the execution of the first backoff function at the end of the time allocated to the RAW group.

## 4. Analytical Model

In this section, we present an analytical model to evaluate the average aggregate throughput of a single-hop IEEE 802.11ah network that is based on a discrete-time Markov chain model. The model describes the backoff operation of a station within its assigned RAW slot. It is assumed that there are *n* stations in the network operating in the basic access mode, i.e., without RTS/CTS control frames, which are evenly distributed among a certain number of RAW slots. Furthermore, we assume ideal channel conditions (i.e., no channel errors) and saturated traffic, i.e., all stations always have a data frame ready for transmission in their buffers. The beacon interval has a fixed length and contains only one RAW group with a fixed duration, i.e., a single RAW group occupies the whole beacon interval. Therefore, the model does not consider the periods when all stations can contend for channel access altogether (in-between RAW groups). The Cross Slot Boundary (CSB) is assumed to be disabled, and a station can transmit multiple data frames in its assigned RAW slot as long as their transmission times fit into the RAW slot. Finally, based on the analytical model, we derive an expression to compute the average aggregate throughput to evaluate network throughput performance.

### 4.1. Markov Model for Backoff Operation

**Figure 3:**
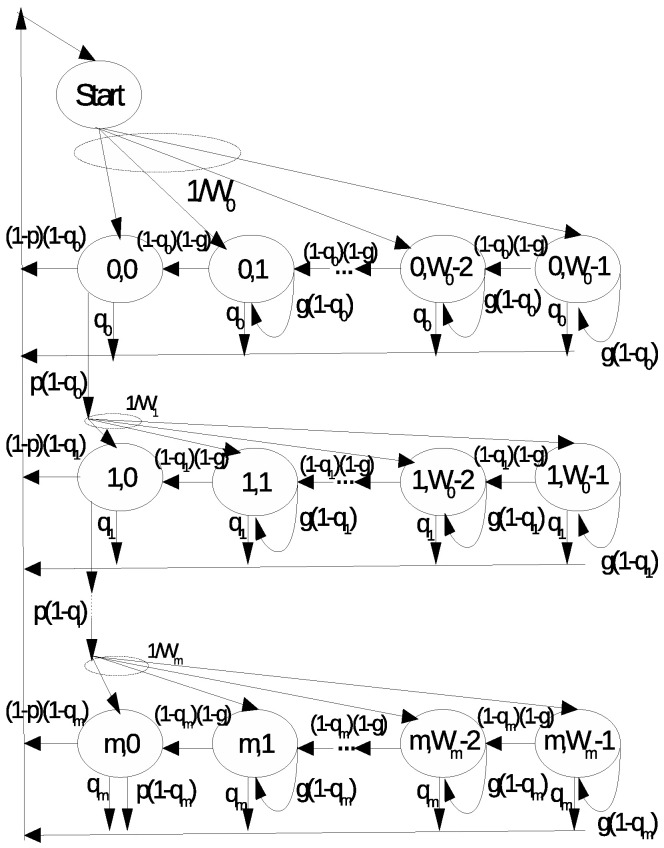
Markov chain model for station operating within its assigned RAW slot according to IEEE 802.11ah.

We model the backoff operation of a station when it is active within its assigned RAW slot according to the two-dimensional discrete-time Markov model shown in Figure 3, where each state represents the backoff counter value and each line represents a backoff stage, i.e., the number of transmission attempts. Similar to the basic IEEE 802.11 DCF, in the beginning of the Markov chain, the station chooses a random backoff value if the channel is sensed idle for a time period greater than or equal to a DIFS time interval, defined in the standard. While the channel is perceived idle, the backoff counter is decremented. Otherwise, the counter is frozen while the channel is sensed busy. The station transmits its data packet when the backoff counter reaches the value 0. If a packet collision occurs, the contention window size is doubled, and the station selects another random value according to Equation (1), moving to the next backoff stage, as long as the number of re-transmission attempts does not exceed the maximum number *m*. If the RAW slot ends in any state, the station returns to the beginning of the Markov chain.

Let b(t) denote the stochastic process that represents the backoff counter value at time *t* for a station assigned to a RAW slot and s(t) denote the stochastic process that represents the backoff stage, i.e., the number of packet transmission attempts so far. Let *p* denote the probability of data packet collision, which is assumed to be constant and independent of the number of transmission attempts. Let *g* denote the “freezing” probability of the backoff counter when the channel is perceived to be busy, according to the DCF mechanism. We assume that it is constant and independent of the backoff stage. Since the RAW slot time can finish at any time during the backoff operation of a station, we represent the occurrence of this event with a transition probability $q_{i}$ departing from every state (i,j),0≤i≤m,0≤j≤Wi−1 and returning to the “start” state. We assume that $q_{i}$ is constant at each stage *i* and the stages are independent of each other. We also assume that the event of RAW slot completion time is independent of the events of freezing the backoff counter value and the occurrence of packet collisions. Finally, let $W_{0}$ denote the minimum contention window size and $W_{i}=2^{i}W_{0}$, 0≤i≤m. Hence, the one-step transition probabilities $P\left\{\left(i_{1},j_{1}\right)|\left(i_{0},j_{0}\right)\right\}=P\left\{s\left(t+1\right)=i_{1},b\left(t+1\right)=j_{1}|s\left(t\right)=i_{0},b\left(t\right)=j_{0}\right\}$ are given by

$$\begin{array}{c} \begin{array}{ccc} P\left\{\left(i,j\right)|\left(i,j+1\right)\right\}=\left(1-q_{i}\right)\left(1-g\right), & i\in [0,m], & j\in [0,W_{i}-1]\\ P\left\{\left(i,j\right)|\left(i,j\right)\right\}=g\left(1-q_{i}\right), & i\in [0,m], & j\in [1,W_{i}-1]\\ P\left\{\left(0,j\right)|\left(i,j\right)\right\}=\frac{q_{i}}{W_{0}}, & i\in [0,m], & j\in [0,W_{i}-1]\\ P\left\{\left(0,j\right)|\left(i,0\right)\right\}=\frac{\left(1-p\right)\left(1-q_{i}\right)}{W_{0}}, & i\in [0,m], & j\in [0,W_{0}-1]\\ P\left\{\left(i+1,j\right)|\left(i,0\right)\right\}=\frac{p(1-q_{i})}{W_{i}}, & i\in [0,m-1], & j\in [0,W_{i}-1]\\ P\left\{\left(0,j\right)|\left(m,0\right)\right\}=\frac{p(1-q_{i})}{W_{0}}, & i=m, & j\in [0,W_{0}-1] \end{array} \end{array}$$

The first equation indicates that the backoff counter advances if the channel is idle and the RAW slot time is not over; the second equation indicates that the backoff counter remains in the same state (frozen) if the channel is busy and the RAW slot time is not over; the third equation contains the probability of ending the RAW slot and return to a new backoff process in the following RAW slot; and the fourth equation represents a successful data frame transmission, which leads to the beginning of a new backoff operation for the next data frame in queue. The fifth equation indicates that a collision has occurred, and the station goes to the next backoff stage; the sixth equation describes the transition probability due to a failed data frame transmission in the last backoff stage: the data frame is discarded and a new backoff operation is initiated for the following data frame in the queue.

### 4.2. Markov Chain Solution

Let $b_{i,j}$ denote the steady-state probability of state (i,j) in the Markov chain, i.e., $b_{i,j}=\lim_{t\to \infty }P\left\{s\left(t\right)=i,b\left(t\right)=j\right\},\;i\in \left[0,m\right],\;j\in \left[0,W_{i}-1\right].$ Using the transition probabilities defined previously, we get

$$\begin{array}{cc} b_{i,j} & =\left\{\begin{array}{cc} B, & i=0,j=W_{0}-1\\ B\times \sum_{l=0}^{W_{0}-\left(j+1\right)}{ [\frac{\left(1-g\right)\left(1-q_{i}\right)}{1-g(1-q_{i})}]}^{l}, & i=0,j\in [0,W_{0}-2]\\ C_{i}, & i\in \left[1,m\right],j=W_{i}-1\\ C_{i}\times \sum_{l=0}^{W_{i}-\left(j+1\right)}{ [\frac{\left(1-g\right)\left(1-q_{i}\right)}{1-g(1-q_{i})}]}^{l}, & i\in \left[1,m\right],j\in \left[1,W_{i}-2\right]\\ \frac{p(1-q_{i-1})}{W_{i}}\times b_{i-1,0}\times { [\frac{\left(1-g\right)\left(1-q_{i}\right)}{1-g(1-q_{i})}]}^{l}, & i\in [1,m],j=0, \end{array}\right. \end{array}$$

where

(2) $$\begin{array}{c} B=\frac{M}{W_{0}\left(1-g\left(1-q_{i}\right)\right)}, \end{array}$$

(3) $$\begin{array}{c} C_{i}=\frac{p(1-q_{i-1})}{W_{i}\left(1-g\left(1-q_{i}\right)\right)}\times b_{i-1,0}, \end{array}$$

and

(4) $$\begin{array}{c} M=\sum_{i=0}^{m}\sum_{j=0}^{W_{i}-1}q_{i}b_{i,j}+ [\left(1-p\right)\sum_{i=0}^{m}\left(1-q_{i}\right)b_{i,0}]+p\left(1-q_{i}\right)b_{m,0}. \end{array}$$

Since a station transmits a packet when it reaches any state $b_{i,0}$, for i∈[0,m], the steady-state packet transmission probability is given by

(5) $$\begin{array}{cc} \tau & =\;\sum_{i=0}^{m}b_{i,0}=\sum_{i=0}^{m}\frac{p(1-q_{i-1})}{W_{i}} [\sum_{l=0}^{W_{i}-1}{ [\frac{\left(1-g\right)\left(1-q_{i}\right)}{1-g(1-q_{i})}]}^{l}\times b_{i-1,0}]. \end{array}$$

If *n* denotes the number of stations competing for the channel in a RAW slot, then a packet collision occurs if one or more of the n−1 remaining stations transmit a packet at the same time. Assuming that all stations transmit packets independently of each other, the packet collision probability *p* will be given by

(6) $$\begin{array}{c} p=1-{\left(1-\tau \right)}^{\left(n-1\right)}. \end{array}$$

Similarly, the probability *g* of a busy channel is the probability of having at least another node transmitting over the channel, i.e.,

(7) $$\begin{array}{c} g=p=1-{\left(1-\tau \right)}^{\left(n-1\right)}. \end{array}$$

The steady-state probabilities of the states of the Markov chain can then be computed by numerical calculation using the normalization condition

(8) $$\begin{array}{c} \sum_{i=0}^{m}\sum_{j=0}^{W_{i}-1}b_{i,j}=1, \end{array}$$

and Equation (5). Finally, we need to compute the probability $q_{i}$ of RAW slot time completion, upon which the station cancels its backoff operation and waits for the next RAW slot in the following beacon interval. Given the complexity in deriving a closed-form expression for the probability of RAW slot time completion $q_{i}$, we extend our previous work [17] by proposing a new heuristic expression for the probability $q_{i}$, which aims to represent this probability more realistically and obtain a more accurate model. The development for the computation of the probability $q_{i}$ is introduced next.

### 4.3. RAW Slot Time Completion Probability

The derivation of the probability of RAW slot time completion is not a trivial task because it depends on a number of events whose characterizations are not easily obtained. Because of that, we adopt a heuristic approach for its computation by considering some key aspects and assumptions. First, we notice that the higher the number of transmission attempts for a given data packet, the longer the time a station spends in backoff and, consequently, the higher the chances of RAW slot time completion during the transmission attempt of that specific data packet. Therefore, for any data packet a station attempts to transmit, we assume that the chances of RAW slot time completion increase in proportion to the number of stages traversed during backoff.

However, during the execution of a given RAW slot, and under traffic saturation, the station may transmit multiple data packets while contending for channel access with other stations assigned to the same RAW slot. Hence, if we assume that the IEEE 802.11ah operation is fair, each of the *n* contending stations should receive an equal “share” of channel access time within a given RAW slot, i.e., each station should get a fraction 1/n of the total RAW slot time for channel access. In this sense, a station is unable to transmit more data packets once its “share” of channel access time is over. As a result, while the station may have performed the backoff algorithm for a number of consecutive data packets, the RAW slot will finish during backoff operation in the transmission attempt of a specific data packet. In other words, such an event determines in which packet transmission attempt the completion of the RAW slot time actually occurs.

Finally, we note that the RAW slot to which the station was assigned occupies a certain amount of time within the beacon interval. Therefore, the ratio between the RAW slot time and the beacon time interval provides an estimate for the probability of finding the station within its RAW slot at any given time. Based on such observations and assumptions, we are interested in the probability of the event of RAW slot time completion during the execution of the *i*-th backoff stage for a given packet, i.e., we are interested in the probability of the joint conditional event $E_{i}=$ {RAW slot ends, STA’s fraction of RAW ends, RAW ends in this backoff stage | stage *i*}, where STA refers to the station performing backoff. From the Markov chain in Figure 3, the probability $q_{i}=P\left\{E_{i}\right\}$ of RAW slot time completion during backoff stage i∈{0,…,m} of attempting a given packet transmission will be given by

(9) $$\begin{array}{cc} q_{i}= & \;P\{\mathrm{RAW}\;\mathrm{slot}\;\mathrm{ends},\;\mathrm{STA}\prime s\;\mathrm{fraction}\;\mathrm{of}\;\mathrm{RAW}\;\mathrm{ends},\mathrm{RAW}\;\mathrm{ends}\;\mathrm{in}\;\mathrm{this}\;\mathrm{stage}|\mathrm{stage}\;i\}, \end{array}$$

which can be rewritten as

(10) $$\begin{array}{cc} q_{i} & =P\{\mathrm{RAW}\;\mathrm{slot}\;\mathrm{ends}|\mathrm{STA}\prime s\;\mathrm{fraction}\;\mathrm{of}\;\mathrm{RAW}\;\mathrm{ends},\mathrm{RAW}\;\mathrm{ends}\;\mathrm{in}\;\mathrm{this}\;\mathrm{stage},\;\mathrm{stage}\;i\}\\ \; & \times P\{\mathrm{STA}\prime s\;\mathrm{fraction}\;\mathrm{of}\;\mathrm{RAW}\;\mathrm{ends}|\mathrm{RAW}\;\mathrm{ends}\;\mathrm{in}\;\mathrm{this}\;\mathrm{stage},\;\mathrm{state}\;i\}\\ \; & \times P\{\mathrm{RAW}\mathrm{ends}\;\mathrm{in}\;\mathrm{this}\;\mathrm{stage}|\mathrm{stage}\;i\}. \end{array}$$

Since the end of a RAW slot does not depend on the operation of any given station (its duration is defined by the access point), we have

(11) $$\begin{array}{cc} \; & P\{\mathrm{RAW}\;\mathrm{slot}\;\mathrm{ends}|\mathrm{STA}\prime s\;\mathrm{fraction}\;\mathrm{of}\;\mathrm{RAW}\;\mathrm{ends},\;\mathrm{RAW}\;\mathrm{ends}\;\mathrm{in}\;\mathrm{this}\;\mathrm{stage},\;\mathrm{stage}\;i\}\\ \; & =P\left\{\mathrm{RAW}\;\mathrm{slot}\;\mathrm{ends}\right\}=1-\frac{\left(T_{slot}-T_{h}-T_{g}\right)}{T_{BI}}, \end{array}$$

where $T_{slot}$, $T_{h}$, $T_{g}$, and $T_{BI}$ are the RAW slot time, the holding time (assuming the CSB is disabled), the guard interval between RAW slots, and the beacon interval, respectively. Note that the shorter the RAW slot time $T_{slot}$ is, the higher the chances of RAW slot time completion. Now, assuming that the end of a station’s fair share of channel access is not dependent on the specific backoff stage the station is currently performing, we have

(12) $$\begin{array}{cc} \; & P\{\mathrm{STA}\prime s\;\mathrm{fraction}\;\mathrm{of}\;\mathrm{RAW}\;\mathrm{ends}|\mathrm{RAW}\;\mathrm{ends}\;\mathrm{in}\;\mathrm{this}\;\mathrm{stage},\;\mathrm{stage}\;i\}\\ \; & =P\left\{\mathrm{STA}\prime s\;\mathrm{fraction}\;\mathrm{of}\;\mathrm{RAW}\;\mathrm{ends}\right\}=1-\frac{1}{n}, \end{array}$$

Since it is assumed that each station occupies 1/n of the RAW slot time $T_{slot}$, at last, for any data packet a station attempts to transmit, the chances of RAW slot time completion are assumed to increase in proportion to the number of backoff stages traversed. Therefore,

(13) $$\begin{array}{cc} \; & P\left\{\mathrm{RAW}\;\mathrm{ends}\;\mathrm{in}\;\mathrm{this}\;\mathrm{stage}|\mathrm{stage}\;i\right\}=\frac{i}{m+1}. \end{array}$$

Finally, from Equations (10)–(13), we obtain

(14) $$\begin{array}{c} q_{i}= [1-\frac{\left(T_{slot}-T_{h}-T_{g}\right)}{T_{BI}}]\left(1-\frac{1}{n}\right)\left(\frac{i}{m+1}\right). \end{array}$$

### 4.4. Throughput Computation

In this section, we compute the average aggregate throughput within a beacon interval, i.e., the sum average throughput across all RAW slots contained in all RAW groups defined within the beacon interval. Different from our previous work [17], the computation of the average aggregate throughput is extended and presented in more detail. First, we explain how the effective throughput for a given RAW slot is computed and, based on that, we show how the overall average aggregate throughput can be computed for a complete set of RAW groups and RAW slots. We assume that only the basic access mechanism is used (i.e., no RTS/CTS frames). Let us first consider the average throughput over a single RAW slot *i*. Using Bianchi’s approach [29], let $n_{i}$ denote the number of stations within RAW slot *i* and $P_{tr}^{i}$ denote the probability that a frame is transmitted over the channel during the RAW slot *i*, i.e., $P_{tr}^{i}=1-{\left(1-\tau^{i}\right)}^{n_{i}}$, and let $P_{s}^{i}$ denote the conditional probability of a successful DATA frame transmission within the RAW slot *i*, given by

(15) $$\begin{array}{c} P_{s^{i}}=\frac{n_{i}\tau^{i}{\left(1-\tau^{i}\right)}^{\left(n_{i}-1\right)}}{P_{tr^{i}}}. \end{array}$$

Hence, the probability of a successful DATA frame transmission is $P_{s}^{i}P_{tr}^{i}$, the probability that a slot is empty is $\left(1-P_{tr}^{i}\right)$, and the probability of DATA collision is $\left(1-P_{s}^{i}\right)P_{tr}^{i}$. If E[P] denotes the average length of a DATA frame payload, the throughput over a given RAW slot *i* is given by

(16) $$\begin{array}{cc} S_{DATA_{i}} & =\frac{P_{s}^{i}P_{tr}^{i}E\left[P\right]}{\left(1-P_{tr}^{i}\right)\sigma +P_{s}^{i}P_{tr}^{i}T_{s}+\left(1-P_{s}^{i}\right)P_{tr}^{i}T_{c}}, \end{array}$$

where σ is the duration of a mini-slot (as defined by the standard), $T_{s}$ is the time the channel is busy due to a successful DATA frame transmission, and $T_{c}$ is the time interval the channel is busy due to collisions. The values of $T_{s}$ and $T_{c}$ are calculated according to

(17) $$\begin{array}{c} T_{s}=\mathrm{DIFS}+\frac{H}{R_{h}}+\frac{E[P]}{R_{d}}+2\delta +\mathrm{SIFS}+\mathrm{ACK} \end{array}$$

and

(18) $$\begin{array}{c} T_{c}=\mathrm{DIFS}+\frac{H}{R_{h}}+\frac{E[P]}{R_{d}}+\mathrm{SIFS}+{\mathrm{ACK}}_{TO}, \end{array}$$

where *H* is the length of the header of a DATA frame, which contains PHY and MAC layer headers, δ is the propagation delay, $R_{h}$ and $R_{d}$ are the transmission rates for the header and payload fields, respectively, ACK and ${ACK}_{TO}$ refer to the duration of the ACK and ACK timeout, and DIFS and SIFS are specified in the IEEE 802.11ah standard.

Now, we are interested in obtaining the effective throughput within a beacon interval corresponding to the data received from stations allocated to RAW slot *i*. In this case, we must take into account the time the stations spend waiting for the end of other RAW slots to which they were not assigned. Note that, from the point of view of the access point, it will receive an amount of data from stations allocated to different RAW slots over a period of time. Therefore, over the duration of a beacon interval, the AP will receive a certain amount of data from stations allocated to RAW slot *i*. Hence, the effective throughput $S_{RAW\_SLOT_{i}}$, over a beacon interval, for stations assigned to RAW slot *i* is

(19) $$\begin{array}{c} \;\;\;\;S_{RAW\_SLOT_{i}}=S_{DATA_{i}}\times \frac{(\mathrm{RAW}\;\mathrm{slot}\;{\mathrm{time})}_{i}-T_{h}-T_{g}}{\mathrm{Beacon}\;\mathrm{Interval}}, \end{array}$$

where $T_{h}=T_{s}$ and $T_{g}$ refer to the holding and guard periods, respectively. The average aggregate throughput $S_{BI}$, in a given beacon interval, can be obtained by adding the effective throughput $S_{RAW\_SLOT_{i}}$ of each RAW slot *i* across all RAW groups *l* in the beacon interval, i.e.,

(20) $$\begin{array}{c} S_{BEACON}=\sum_{l=1}^{r}\sum_{i=0}^{s_{l}}S_{RAW\_SLOT_{i}}, \end{array}$$

where $s_{l}$ is the number of RAW slots within each RAW group *l*, and *r* is the number of RAW groups within the beacon interval. In this work, we consider r=1, i.e., the scenario of a single RAW group in a beacon interval. As a final note, Equation (20) assumes that there are no free access periods between RAW groups.

## 5. Analytical Models Used in Comparison

In this section, we briefly present the main features of two analytical models available in the literature that we use to compare with our model. From Section 2, we can identify two main lines of work: those that follow a mean value analysis, as originally used by Zheng et al. [20], and others that propose Markov chain models. In this second group, however, there are works that adopt Bianchi’s [29] original model without considering any specific features of the IEEE 802.11ah standard. Therefore, we selected Sangeetha et al. model [21] due to their effort to represent some of the key characteristics of IEEE 802.11ah. In addition, Zheng’s and Sangeetha’s works make different assumptions regarding protocol behavior, and they treat the event of RAW slot time completion differently, which is key for comparison with our model.

First, we present the model introduced by Zheng et al. [20], who used mean value analysis (MVA) to study the efficiency of the RAW mechanism in single-hop networks. In their work, they denote the RAW mechanism of the IEEE 802.11ah as the Group-Synchronized Distributed Coordination Function (GS-DCF). We chose Zheng’s model to compare with our model because it is one of the first works on IEEE 802.11ah, and many papers have used GS-DCF to model the RAW mechanism. Another work we use for performance comparison is the one by Sangeetha et al. [21]. Their work is based on Bianchi’s approach to model the operation of a station according to a discrete-time Markov chain, based on which they derive the average throughput and delay performance of IEEE 802.11ah single-hop networks under the RAW-based channel access mechanism. Among the reasons to choose Sangeetha’s model for performance comparison, we mention the fact that they adopt a modeling approach similar to ours, but it is different in terms of assumptions regarding protocol behavior, and the fact that it is a recent work on the analytical modeling of IEEE 802.11ah. It is also worth mentioning that both analytical models make similar assumptions as ours, such as saturated traffic and ideal channel conditions, and they consider that stations are equally divided among RAW slots.

### 5.1. Zheng et al. Analytical Model

Zheng et al. [20] use a totally different approach from ours to model IEEE 802.11ah networks. Their model is based on mean value analysis to obtain the conditional packet collision probability *p*. According to their model, given that a station has a data frame ready for transmission, the probability τ to transmit it in an idle slot is given by

(21) $$\begin{array}{c} \tau =\frac{E[R]}{{E[B]+E[R]}^{\prime }} \end{array}$$

where E[B] and E[R] are the average number of backoff mini-slots and transmission attempts experienced by one packet, respectively. For each packet, the number of transmission attempts follows a truncated geometric distribution with success probability (1−p). Thus, E[B] and E[R] can be obtained as follows:

(22) $$\begin{array}{cc} E[R] & = [\sum_{r=1}^{R_{max}}r\left(1-p\right)p^{r-1}]+R_{max}p^{R_{max}-1}=\sum_{r=1}^{R_{max}}p^{r-1} \end{array}$$

and

(23) $$\begin{array}{cc} E[B] & =\frac{1}{2}\sum_{r=1}^{R_{max}}\min\left\{2^{r-1}CW_{min},CW_{max}\right\}\left(1-p\right)p^{r-1}\\ \; & =\frac{1}{2}\sum_{r=1}^{R_{max}}\min\left\{2^{r-1}CW_{min},CW_{max}\right\}p^{r-1}, \end{array}$$

where $R_{max}$ is the number of packet transmission attempts before the packet is dropped.

It is important to note that this model does not consider the periods of time when the backoff counter is frozen due to channel activity. However, similar to our approach, a collision happens if more than one station transmits in the same slot. Thus, the conditional collision probability *p* is given by

(24) $$\begin{array}{c} p=1-{\left(1-\tau \right)}^{\left(g-1\right)}, \end{array}$$

where *g* is the number of stations in a RAW slot. Then, assuming that the number of backoff mini-slots a station has to traverse before transmitting follows a geometric distribution, and if $n_{b}$ denotes the number of backoff mini-slots a station waits for its TXOP, they obtained

(25) $$\begin{array}{c} \mathrm{Prob}\left\{n_{b}=j\right\}=\tau {\left(1-\tau \right)}^{j-1}, \end{array}$$

where j≥1. Let $T_{b}$ denote the duration of the backoff counters, in mini-slots, between consecutive TXOPs in a RAW slot. $T_{b}$ is equal to the minimum backoff counter duration value among all *g* STAs’ backoff counters in the RAW slot, and it also follows a geometric distribution with parameter $q^{\prime }=1-\left(1-\tau \right)g$. Hence,

(26) $$\begin{array}{cc} P_{T_{b}|G}\left(j|g\right) & =q^{\prime }{\left(1-q^{\prime }\right)}^{j-1},\;j\geq 1,\;g\geq 2. \end{array}$$

Based on Equations (25) and (26), they derived the distribution for the duration of multiple transactions in a RAW slot and the distribution for the number of transactions from all stations in a given RAW slot. Finally, the throughput expression in a given RAW slot was given by

(27) $$\begin{array}{c} Th\left(g\right)=\frac{L}{T_{R}}E_{M|G}\left(g\right)P_{suc|G}\left(1|g\right), \end{array}$$

where *L* is the packet transmission time, $T_{R}$ is the total duration of a RAW group, *M* is the random variable that indicates the number of transactions within a RAW slot, $E_{M|G}\left(g\right)$ is the expected number of transactions in the RAW slot, and $P_{suc|G}\left(1|g\right)$ is the probability of a successful transmission in a RAW group, following Bianchi’s approach. We refer the reader to [20] for the complete throughput expression and further details. Their model was validated based on simulation results derived from a simulator designed by themselves. Their analysis compared their results with the standard IEEE 802.11 DCF, and they showed the benefits of using the grouping scheme in dense networks, as opposed to allowing all stations to have access to the channel altogether.

The main difference between Zheng’s model and ours is that, when using the mean value analysis approach, Zheng’s model computes the average number of transactions, i.e., packet transmissions within a RAW slot and the average duration of these transactions, to obtain their results. However, this approach does not include the event of freezing backoff when the channel is perceived busy. Furthermore, their model assumes that a station saves its backoff counter value when the RAW slot ends and resumes its backoff operation in the next assigned RAW slot. In reality, in the beginning of a RAW slot, the backoff function is renewed for all the STAs assigned to that particular RAW slot. In our model, a station discards its backoff counter at the end of a RAW slot and starts a new backoff process in the beginning of its next assigned slot.

### 5.2. Sangeetha et al. Analytical Model

Sangeetha et al. [21] modeled the behavior of a station in its backoff process within a RAW slot. They proposed an analytical model similar to ours, based on a discrete-time Markov chain, and used Bianchi’s approach for throughput computation. Similar to Zheng’s model, Sangeetha et al. assume that, at the end of a RAW slot, the station stores the value of the backoff counter in which it has stopped, and it resumes its backoff operation from that saved value in the beginning of its next RAW slot (the one it was assigned to). Because of their assumption, the backoff counter is frozen with probability 1 when the station is outside its designed RAW slot. According to their analytical model, the probability that the backoff is frozen inside the RAW slot *i* is

(28) $$\begin{array}{c} p_{f,i}=\frac{T_{a,i}}{T_{R}} [1-{\left(1-\tau_{i}\right)}^{n_{i}-1}]+\frac{T_{R}-T_{a,i}}{T_{R}}, \end{array}$$

where $T_{a,i}$ is the available access period for STAs within RAW slot *i*, $T_{R}$ is the total duration of the RAW group period, $\tau_{i}$ is the transmission probability in the RAW slot *i*, and *n* is the number of stations within RAW slot *i*. When the RAW slot ends, the station remains in the same backoff state with probability 1 and continues the backoff process in the next assigned RAW slot.

A defer state is also defined in the Markov chain to represent the idea that if the backoff counter reaches the value 1, it only decrements to zero if there is sufficient time for a successful packet transmission. Otherwise, the station goes to the *defer state* with probability

(29) $$\begin{array}{c} p_{d,i}=\left\{\begin{array}{cc} \frac{T_{s}}{T_{a,i}}, & T_{s}\leq T_{a,i}\\ 1, & \mathrm{otherwise}, \end{array}\right. \end{array}$$

where $T_{s}$ is the duration of a successful transmission. They presented the Markov chain solution and provided a throughput computation based on Bianchi’s approach. They evaluated the average throughput and packet delay performances and validated their model with simulations in the ns-3 simulator, according to the PHY and MAC layer parameters of IEEE 802.11 and IEEE 802.11ah.

The main differences between our model and Sangeetha’s are the same as those we pointed out regarding Zheng’s model, i.e., the way they handle the event of RAW slot time completion. In both cases, they modify the backoff process that is originally defined in the standard and simplify it by assuming that the station saves the value of its backoff counter at the time the RAW slot ends, and it resumes its operation at the same backoff counter value at the beginning of its next assigned RAW slot. Differently from them, we model the actual backoff process as defined by the standard, where a station must discard the backoff counter value in which the RAW slot completion happens and initiates a new backoff process in the beginning of is next assigned RAW slot.

## 6. Numerical Results

In this section, we present numerical results derived from our analytical model and compare them with computer simulations carried out in the ns-3 simulator based on the IEEE 802.11ah module previously developed by Le Tian et al. [19]. We focus on single-hop networks under ideal channel conditions and saturated traffic (i.e., all nodes always have a data frame ready for transmission in their queue). In the proposed scenario, each beacon interval has a fixed duration and contains only one RAW group with a given number of RAW slots, each with the same and fixed time duration. The RAW group, with its associated set of RAW slots, occupies the whole beacon interval. Every beacon interval has the same time duration, and the stations are evenly distributed among a given number of RAW slots. We present results for the average aggregate throughput by considering different numbers of stations and RAW slots per beacon interval. Table 1 and Table 2 show the parameter values used in all scenarios. The parameter values were chosen based on the IEEE 802.11ah standard [13] and on the simulation study carried out by Le Tian et al. [19] to validate their IEEE 802.11ah module developed for the ns-3 simulator.

In Section 6.1, we first present an investigation about the impact of the proposed probability of RAW slot time completion ($q_{i}$ in our analytical model) on the accuracy of the numerical results with respect to the average aggregate throughput obtained via discrete-event simulations on the ns-3 simulator. This set of results is important to establish the key role that this probability plays in the accuracy of the proposed analytical model. Then, in Section 6.2, we present a performance comparison of predicted average aggregate throughput between our analytical model and the two other analytical models previously discussed, adopting the results derived by simulations performed on the ns-3 simulator based on the IEEE 802.11ah module developed by Le Tian et al. [19] as a benchmark.

### 6.1. Impact of RAW Slot Time Completion Probability on Throughput

In order to understand the impact of the proposed probability of RAW slot time completion on the accuracy of the analytical model, we compare the average aggregate throughput computed in two cases: when the probability of RAW slot time completion is either present or absent in the Markov Chain model of Figure 3 (i.e., we consider the cases $q_{i}=0$ or $q_{i}\neq 0,\;\forall i\in \left\{0,\ldots ,m\right\}$). In this section, we compute the average aggregate throughput according to Equation (20) for the cases of 2, 5, and 10 RAW slots per beacon interval and vary the total number of stations in the network from 5 to 100 nodes, evenly distributed among the RAW slots (note that, depending on the number of stations, there can be some RAW slots with fewer or no stations).

**Figure 4:**
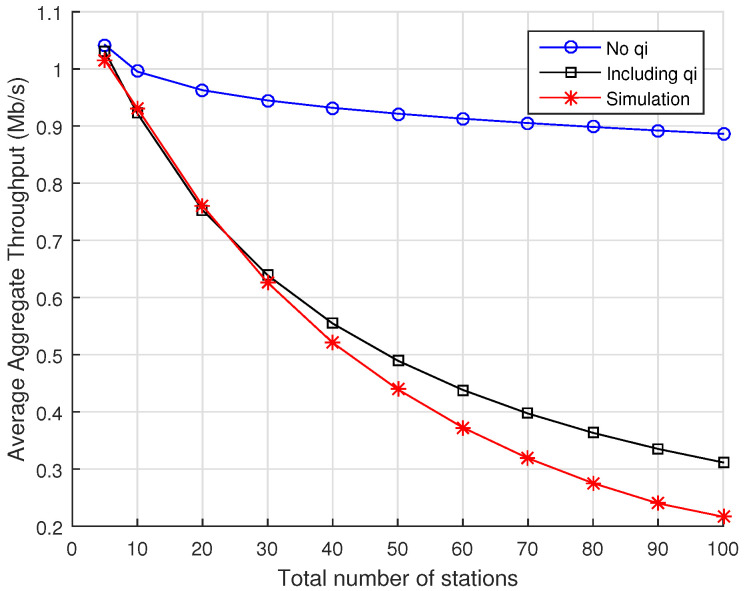
Average aggregate throughput as a function of the total number of stations divided into 2 RAW slots within a single RAW group per beacon interval. Numerical results for the analytical model are displayed for the cases when either Equation (14) or q=0 are considered for the probability of RAW slot time completion, along with the results obtained with ns-3 simulations.

Figure 4 contains the results for the case when there are only 2 RAW slots within the RAW group. The blue line with circles contains the results for the case when the probability of RAW slot time completion is absent from the model, while the black curve with squares shows the results for the case when the probability of RAW slot time completion is taken into account in the model. The results from ns-3 simulations are shown in red line with asterisks. As we can see, the results derived from the analytical model without considering the probability of RAW slot time completion differs significantly from simulations, presenting very optimistic values compared to the case when such probability is considered. For purposes of comparison, the *root-mean-square error* (RMSE) computed between simulation results and the values predicted by the analytical model if the probability of RAW slot time completion is *disregarded* is an RMSE of 0.4160, whereas if such probability is considered, we obtain an RMSE of 0.0471.

**Figure 5:**
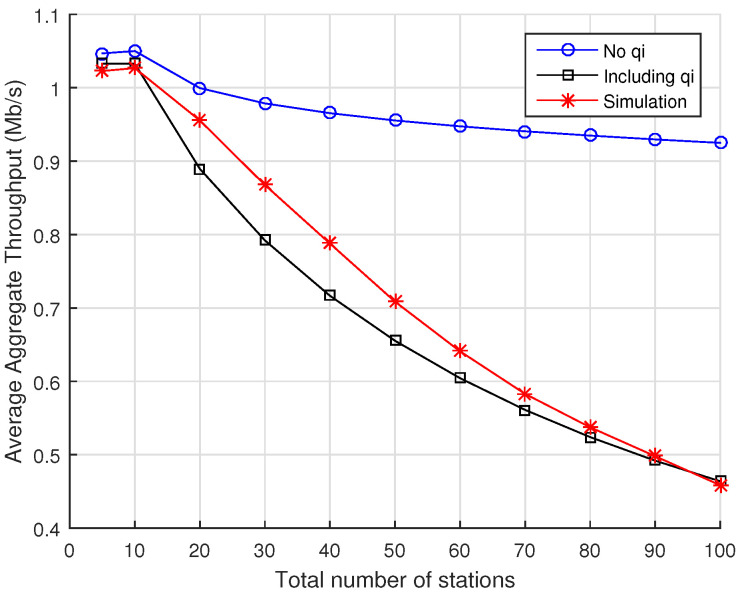
Average aggregate throughput as a function of the total number of stations divided into 5 RAW slots within a single RAW group per beacon interval. Numerical results for the analytical model are displayed for the cases when either Equation (14) or q=0 are considered for the probability of RAW slot time completion, along with the results obtained with ns-3 simulations.

Figure 5 shows the results when there are 5 RAW slots per beacon interval. Again, we observe the strong disagreement with simulation results when the probability of RAW slot time completion is disregarded. In this case, we obtain an RMSE of 0.2352 if $q_{i}=0$, ∀i∈{0,…,m}, while an RMSE of 0.0178 is found if the probability of RAW slot time completion is considered.

**Figure 6:**
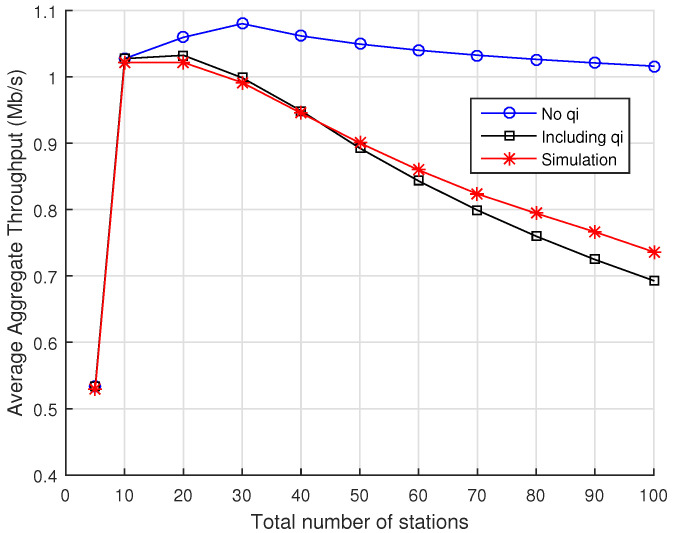
Average aggregate throughput as a function of the total number of stations divided into 10 RAW slots within a single RAW group per beacon interval. Numerical results for the analytical model are displayed for the cases when either Equation (14) or q=0 are considered for the probability of RAW slot completion time, along with the results obtained with ns-3 simulations.

Figure 6 depicts the results for the case when there are 10 RAW slots per beacon interval. As the number of stations per RAW slot decreases, we observe higher aggregate throughput values, since contention decreases at each RAW slot. In this case, an RMSE of 0.1416 is found for the case when there is no probability of RAW slot time completion, and an RMSE of 0.0124 is found if such probability is considered in the analytical model.

### 6.2. Comparison with Other Analytical Models

**Figure 7:**
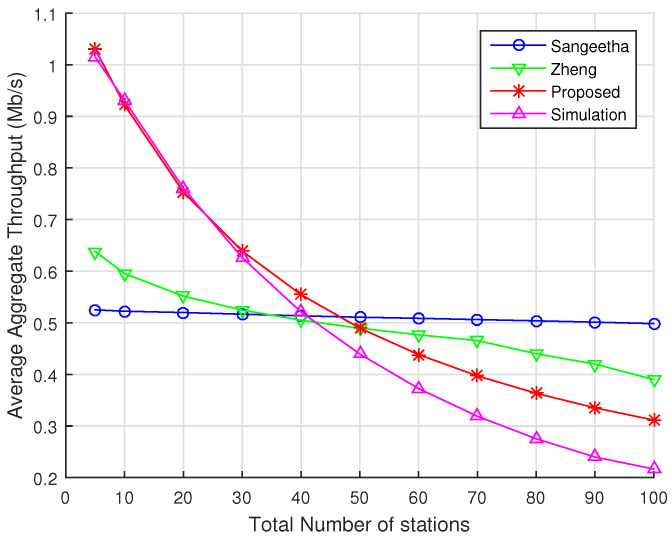
Average aggregate throughput as a function of the total number of stations divided into 2 RAW slots within a single RAW group per beacon interval. Numerical results for our proposed model, Zheng’s model, and Sangeetha’s model are displayed along with the results obtained with ns-3 simulations.

In this section, we compare the average aggregate throughput predicted by our analytical model with those provided by Sangeetha [21] and Zheng [20] models, considering ns-3 simulations as a benchmark. Figure 7 depicts the results for the average aggregate throughput when there are only 2 RAW slots within a beacon interval. As we can observe, Zheng’s and Sangeetha’s models are in strong disagreement with the results obtained via ns-3 simulations. In particular, we observe that Sangeetha’s model predicts almost no throughput decay as the total number of stations increases. In fact, this is in line with what is shown in their original work, which presents similar behavior. For Sangeetha’s model, we obtain an RMSE of 0.258. In the case of Zheng’s model, we observe a higher decay in throughput values as the number of stations increases compared to Sangeetha’s model. However, it does not show much agreement with simulations, with the exception of some specific results (e.g., when the number of stations is 40 and 50). For Zheng’s model we obtain an RMSE of 0.198. As discussed in Section 5.1 and Section 5.2, both models assume that the backoff process is not renewed at the beginning of a new RAW slot, i.e., the stations freeze their backoff counter values at the end of a RAW slot and resume them in the beginning of the next RAW slot. Such feature has a major impact on their overall results. In contrast, our analytical model delivers an RMSE of 0.0471, as previously discussed.

**Figure 8:**
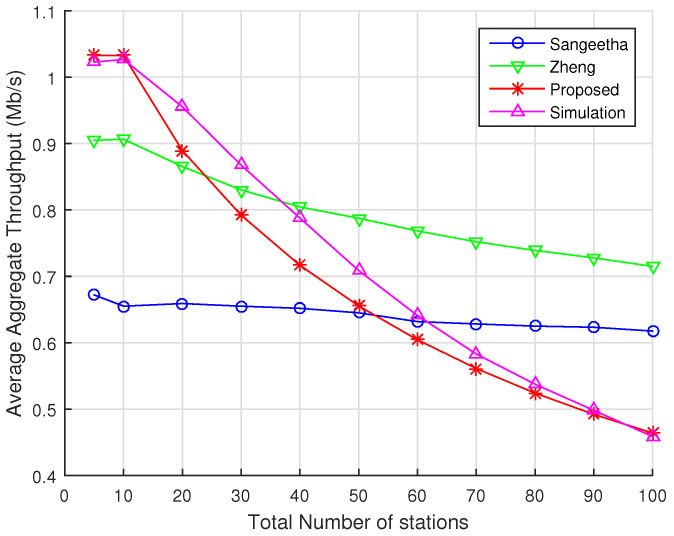
Average aggregate throughput as a function of the total number of stations divided into 5 RAW slots within a single RAW group per beacon interval. Numerical results for our proposed model, Zheng’s model, and Sangeetha’s model are displayed along with the results obtained with ns-3 simulations.

Figure 8 contains the results for the case of 5 RAW slots within a single RAW group in the beacon interval. As expected, the average aggregate throughput increases with respect to the case with 2 RAW slots across all analytical models and simulations because channel contention decreases within each RAW slot and, therefore, more data packets can be transmitted per RAW slot. Sangeetha’s model continues to present a behavior similar to that obtained for 2 RAW slots, but with higher average throughput values and an RMSE of 0.206. Zheng’s model continues to show the trending behavior of throughput decay as the total number of stations increases. However, it still shows considerable discrepancies with simulations, with an RMSE of 0.150. For our model, the results are closer to simulations but slightly more pessimistic in terms of throughput, especially when the number of stations is between 20 and 60. In this scenario, our model delivers an RMSE of 0.0178, which means that Zheng’s RMSE is about 287% higher than ours.

**Figure 9:**
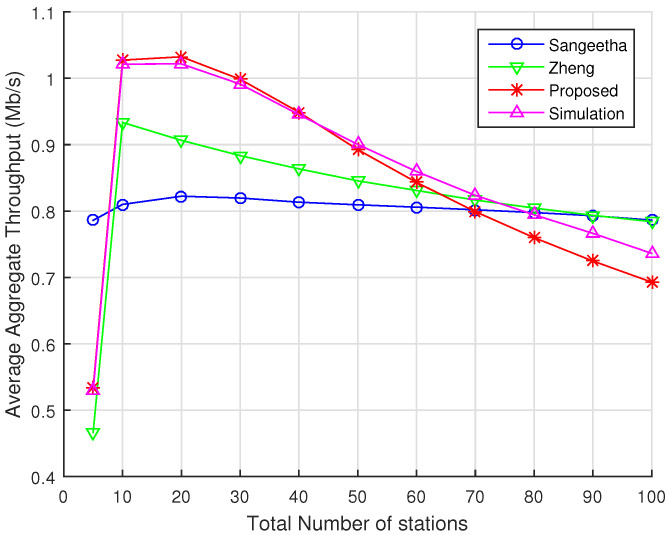
Average aggregate throughput as a function of the total number of stations divided into 10 RAW slots within a single RAW group per beacon interval. Numerical results for our proposed model, Zheng’s model, and Sangeetha’s model are displayed along with the results obtained with ns-3 simulations.

Figure 9 contains the results for the case of 10 RAW slots within a single RAW group in each beacon interval. Note that, when n=5, there are empty RAW slots in the beacon interval, which leads to low throughput values for the three analytical models and simulations. Similar to the other scenarios, our analytical model delivers the closest behavior to simulations, with an RMSE of 0.0124. In this case, however, the predicted throughput values are a bit more pessimistic than simulations, as the number of nodes increases. Zheng’s model presents the general behavior of throughput decay as the number of stations increases. However, while it is more pessimistic than simulations for lower number of stations, it becomes more optimistic as the number of nodes increases, especially for n≥80. Zheng’s model gives an RMSE of 0.068. Finally, Sangeetha’s model presents the same subtle decrease in throughput as the number of stations increases, as observed in the numerical results in their work. In this case, we get an RMSE of 0.139.

## 7. Conclusions

In this paper, we introduced a discrete-time Markov chain model to evaluate the average aggregate throughput performance of an IEEE 802.11ah network under ideal channel conditions and saturated traffic. The model addresses the dynamics of a station within its assigned RAW slot and includes the impact of RAW slot time completion as a key parameter, for which we present a heuristic approach for its derivation and showed its impact on model accuracy through comparisons with computer simulations. The performance of the proposed analytical model was compared to two other analytical models available in the literature, whose numerical results were also compared to computer simulations carried out independently in the ns-3 simulator. The results showed that the proposed analytical model predicts network performance with higher accuracy than the other models (expressed in terms of RMSE).

## 8. Future Works

While there are a number of works that have investigated the performance of IEEE 802.11ah networks under different scenarios regarding the size of the network, type of traffic, and configuration of key parameters, such as the number and size of RAW slots and RAW groups, most of the previous work has focused on static scenarios. In other words, the majority of the previous work assumes that stations are already associated/authenticated to a given access point and do not change location with time (i.e., there is no mobility). Consequently, most previous work does not handle the arrival/departure of a station to/from an IEEE 802.11ah network or the real-time allocation (or re-arrangement) of stations to new RAW slots or RAW groups as a result of their movement or channel state conditions. This is very important because some IoT applications target mobile devices (e.g., robots, UAVs, industrial machines, etc.), which require special attention, especially under dynamic channel conditions: the channel state experienced by a group of static stations is likely to be very different from the channel conditions perceived by mobile stations. Moreover, the mobility of some stations may affect the performance of other stations due to the well-known hidden/exposed terminal problem, which needs to be handled during the re-allocation of RAW slots and RAW groups. Given the importance of such a problem, our research group is currently working on solutions for dynamic scenarios, especially regarding new algorithms for re-allocation of stations to new RAW groups and/or RAW slots with the goal of achieving higher network performance.

Another main challenge in the deployment of IoT applications is the fact that most wireless devices are powered with conventional batteries, which typically require frequent replacements. As IoT applications are diverse, ranging from healthcare to agriculture and industrial environments, the connected devices can be located in places that are difficult to access, where maintenance and the frequent replacement of batteries make the operation of such devices inefficient and expensive. In addition, billions of batteries are expected to be discarded every year, which has a negative impact on the environment. A promising solution to address this issue is to integrate radio-frequency (RF) energy harvesting technologies into IoT systems, which is a more environmentally friendly solution that can extend the lifetime of IoT devices and even replace battery power completely, in some cases [45,46]. However, this is a challenging integration due to the characteristics of energy harvesting technologies and the different power requirements of IoT devices. Although there are different approaches in the literature for modifications in MAC-layer protocols to reduce energy consumption, there is still a gap to understand possible MAC improvements to enable RF energy harvesting in IoT systems and ensure the energy self-sustainability of the network [47]. Moreover, to date, most works have tackled the introduction of RF energy harvesting only in traditional Wi-Fi networks [48,49,50,51], and only recently has there been some effort to introduce energy harvesting into IEEE 802.11ah networks [36]. Therefore, there is still a need to address RF energy harvesting in IEEE 802.11ah networks and, because of that, our future work aims to integrate RF energy harvesting in the operation of IEEE 802.11ah networks. For that, we intend to exploit its RAW and *target wake-up time* (TWT) mechanisms in the design of innovative grouping strategies that can deliver a more sustainable and energy-efficient operation of the network.

## Figures and Tables

**Table 1:** Values of PHY-layer parameters used in simulations and numerical results.

Parameter	Value
Basic rate	1 Mb/s
Data rate	7.8 Mb/s
Channel bandwidth	2 MHz
Modulation and coding scheme	MCS8
PHY layer header	192μs
Slot duration (σ)	52μs
Propagation delay (δ)	3.3μs

**Table 2:** Values of MAC-layer and other parameters used in simulations and numerical results.

Parameter	Value
SIFS	160μs
DIFS	264μs
$ACK_{TO}$	2×δ + SIFS + ACK
$CW_{min}$ / $CW_{max}$	16/1024
Payload size	256 bytes
ACK	14 bytes + PHY Header
MAC layer header	34 bytes
Beacon interval	0.1 s
Guard interval ($T_{g}$)	8μs
Holding period ($T_{h}$)	$T_{s}$

## Data Availability

Not applicable.

## References

[1] Al-Fuqaha A., Guizani M., Mohammadi M., Aledhari M., Ayyash M. (2015). Internet of things: A survey on enabling technologies, protocols, and applications. IEEE Commun. Surv. Tutor..

[2] Minoli D., Sohraby K., Occhiogrosso B. (2017). IoT Considerations, Requirements, and Architectures for Smart Buildings—Energy Optimization and Next-Generation Building Management Systems. IEEE Internet Things J..

[3] Yaqoob I., Ahmed E., Hashem I.A.T., Ahmed A.I.A., Gani A., Imran M., Guizani M. (2017). Internet of Things Architecture: Recent Advances, Taxonomy, Requirements, and Open Challenges. IEEE Wirel. Commun..

[4] Sisinni E., Saifullah A., Han S., Jennehag U., Gidlund M. (2018). Industrial internet of things: Challenges, opportunities, and directions. IEEE Trans. Ind. Inform..

[5] Bellini P., Nesi P., Pantaleo G. (2022). IoT-enabled smart cities: A review of concepts, frameworks and key technologies. Appl. Sci..

[6] Quy V.K., Hau N.V., Anh D.V., Quy N.M., Ban N.T., Lanza S., Randazzo G., Muzirafuti A. (2022). IoT-Enabled Smart Agriculture: Architecture, Applications, and Challenges. Appl. Sci..

[7] Misra N.N., Dixit Y., Al-Mallahi A., Bhullar M.S., Upadhyay R., Martynenko A. (2022). IoT, Big Data, and Artificial Intelligence in Agriculture and Food Industry. IEEE Internet Things J..

[8] Kassab W., Darabkh K.A. (2020). A–Z survey of Internet of Things: Architectures, protocols, applications, recent advances, future directions and recommendations. J. Netw. Comput. Appl..

[9] Guo F., Yu F.R., Zhang H., Li X., Ji H., Leung V.C.M. (2021). Enabling Massive IoT Toward 6G: A Comprehensive Survey. IEEE Internet Things J..

[10] Shen X., Liao W., Yin Q. (2022). A Novel Wireless Resource Management for the 6G-Enabled High-Density Internet of Things. IEEE Wirel. Commun..

[11] Lopez-Perez D., Garcia-Rodriguez A., Galati-Giordano L., Kasslin M., Doppler K. (2019). IEEE 802.11be Extremely High Throughput: The Next Generation of Wi-Fi Technology Beyond 802.11ax. IEEE Commun. Mag..

[12] Chaudhari B.S., Zennaro M., Borkar S. (2020). LPWAN Technologies: Emerging Application Characteristics, Requirements, and Design Considerations. Future Internet.

[13] (2016). Amendment to IEEE Std 802.11-2016, as amended by IEEE Std 802.11ai-2016; IEEE Standard for Information technology—Telecommunications and information exchange between systems—Local and metropolitan area networks–Specific requirements—Part 11: Wireless LAN Medium Access Control (MAC) and Physical Layer (PHY) Specifications Amendment 2: Sub 1 GHz License Exempt Operation.

[14] Khorov E., Lyakhov A., Krotov A., Guschin A. (2015). A survey on IEEE 802.11 ah: An enabling networking technology for smart cities. Comput. Commun..

[15] Tian L., Santi S., Seferagić A., Lan J., Famaey J. (2021). Wi-Fi HaLow for the Internet of Things: An up-to-date survey on IEEE 802.11ah research. J. Netw. Comput. Appl..

[16] Ahmed N., De D., Barbhuiya F.A., Hussain M.I. (2022). MAC Protocols for IEEE 802.11ah-Based Internet of Things: A Survey. IEEE Internet Things J..

[17] Soares S.M., Carvalho M.M. Throughput Analytical Modeling of IEEE 802. Proceedings of the 16th IEEE Annual Consumer Communications & Networking Conference.

[18] Nsnam (2016). ns-3|A Discrete-Event Network Simulator for Internet Systems. http://www.nsnam.org.

[19] Tian L., Deronne S., Latré S., Famaey J. Implementation and Validation of an IEEE 802.11ah Module for ns-3. Proceedings of the 8th Workshop on ns-3. ACM.

[20] Zheng L., Ni M., Cai L., Pan J., Ghosh C., Doppler K. (2014). Performance Analysis of Group-Synchronized DCF for Dense IEEE 802.11 Networks. IEEE Trans. Wirel. Commun..

[21] Sangeetha U., Babu A. (2019). Performance Analysis of IEEE 802.11ah Wireless Local Area Network Under the Restricted Access Window-Based Mechanism. Int. J. Commun. Syst..

[22] Ali M.Z., Mišić J., Mišić V.B. (2019). Performance Evaluation of Heterogeneous IoT Nodes with Differentiated QoS in IEEE 802.11ah RAW Mechanism. IEEE Trans. Veh. Technol..

[23] Tian L., Šljivo A., Santi S., De Poorter E., Hoebeke J., Famaey J. Extension of the IEEE 802.11ah ns-3 Simulation Module. Proceedings of the 10th Workshop on ns-3.

[24] Qutab-ud din, Muhammad H.A., Badihi B., Larmo A., Torsner J., Valkama M. Performance Analysis of IoT-Enabling IEEE 802.11ah Technology and its RAW Mechanism with Non-cross Slot Boundary Holding Schemes. Proceedings of the IEEE International Symposium on a World of Wireless, Mobile and Multimedia Networks.

[25] Mahesh M., Harigovindan V. (2019). Restricted Access Window-Based Novel Service Differentiation Scheme for Group-Synchronized DCF. IEEE Commun. Lett..

[26] Raeesi O., Pirskanen J., Hazmi A., Levanen T., Valkama M. Performance Evaluation of IEEE 802.11 ah and its Restricted Access Window Mechanism. Proceedings of the IEEE International Conference on Communications Workshops.

[27] Raeesi O., Pirskanen J., Hazmi A., Talvitie J., Valkama M. Performance Enhancement and Evaluation of IEEE 802.11ah Multi-access Point Network Using Restricted Access Window Mechanism. Proceedings of the IEEE International Conference on Distributed Computing in Sensor Systems.

[28] Park C.W., Hwang D., Lee T.J. (2014). Enhancement of IEEE 802.11ah MAC for M2M Communications. IEEE Commun. Lett..

[29] Bianchi G. (2000). Performance Analysis of the IEEE 802.11 Distributed Coordination Function. IEEE J. Sel. Areas Commun..

[30] Sangeetha U., Babu A. (2021). Service Differentiation in IEEE 802.11 ah WLAN under Restricted Access Window based MAC protocol. Comput. Commun..

[31] Ali M.Z., Mišić J., Mišić V.B. Efficiency of restricted access window scheme of IEEE 802.11ah under non-ideal channel condition. Proceedings of the 2018 IEEE International Conference on Internet of Things (iThings) and IEEE Green Computing and Communications (GreenCom) and IEEE Cyber, Physical and Social Computing (CPSCom) and IEEE Smart Data (SmartData).

[32] Badarla S.P., Harigovindan V. (2021). Restricted Access Window-Based Resource Allocation Scheme for Performance Enhancement of IEEE 802.11ah Multi-Rate IoT Networks. IEEE Access.

[33] Khorov E., Krotov A., Lyakhov A. Modelling Machine Type Communication in IEEE 802.11ah Networks. Proceedings of the IEEE International Conference on Communication Workshop.

[34] Khorov E., Lyakhov A., Yusupov R. Two-Slot Based Model of the IEEE 802.11ah Restricted Access Window with Enabled Transmissions Crossing Slot Boundaries. Proceedings of the 2018 IEEE 19th International Symposium on" A World of Wireless, Mobile and Multimedia Networks"(WoWMoM).

[35] Khorov E., Krotov A., Lyakhov A., Yusupov R., Condoluci M., Dohler M., Akyildiz I. (2019). Enabling the Internet of Things With Wi-Fi Halow—Performance Evaluation of the Restricted Access Window. IEEE Access.

[36] Bankov D., Khorov E., Lyakhov A., Famaey J. (2020). Resource Allocation for Machine-Type Communication of Energy-Harvesting Devices in Wi-Fi Halow Networks. Sensors.

[37] Nawaz N., Hafeez M., Zaidi S.A.R., McLernon D.C., Ghogho M. Throughput Enhancement of Restricted Access Window for Uniform Grouping Scheme in IEEE 802.11ah. Proceedings of the IEEE International Conference on Communications.

[38] Lakshmi L.R., Sikdar B. Fair Scheduling in IEEE 802.11 ah Networks for Internet of Things Applications. Proceedings of the 2019 IEEE Global Communications Conference (GLOBECOM).

[39] Kai C., Zhang J., Zhang X., Huang W. (2019). Energy-Efficient Sensor Grouping for IEEE 802.11ah Networks with Max-Min Fairness Guarantees. IEEE Access.

[40] Chang T.C., Lin C.H., Lin K.C.J., Chen W.T. Load-balanced Sensor Grouping for IEEE 802.11ah Networks. Proceedings of the IEEE Global Communications Conference.

[41] Dong M., Wu Z., Gao X., Zhao H. An Efficient Spatial Group Restricted Access Window Scheme for IEEE 802.11ah Networks. Proceedings of the Sixth International Conference on Information Science and Technology.

[42] Yoon S.G., Seo J.O., Bahk S. (2016). Regrouping Algorithm to Alleviate the Hidden Node Problem in 802.11ah Networks. Comput. Netw..

[43] Tian L., Khorov E., Latré S., Famaey J. (2017). Real-time Station Grouping Under Dynamic Traffic for IEEE 802.11ah. Sensors.

[44] Tian L., Mehari M., Santi S., Latré S., De Poorter E., Famaey J. IEEE 802.11ah Restricted Access Window Surrogate Model for Real-time Station Grouping. Proceedings of the IEEE 19th International Symposium on “A World of Wireless, Mobile and Multimedia Networks”.

[45] Huang J., Zhou Y., Ning Z., Gharavi H. (2019). Wireless Power Transfer and Energy Harvesting: Current Status and Future Prospects. IEEE Wirel. Commun..

[46] Sanislav T., Mois G.D., Zeadally S., Folea S.C. (2021). Energy Harvesting Techniques for Internet of Things (IoT). IEEE Access.

[47] Famitafreshi G., Afaqui M.S., Melià-Seguí J. (2021). A Comprehensive Review on Energy Harvesting Integration in IoT Systems from MAC Layer Perspective: Challenges and Opportunities. Sensors.

[48] Ha T., Kim J., Chung J.M. (2017). HE-MAC: Harvest-then-transmit based modified EDCF MAC protocol for wireless powered sensor networks. IEEE Trans. Wirel. Commun..

[49] Lee H., Kim Y., Ahn J.H., Chung M.Y., Lee T.J. (2017). Wi-Fi and wireless power transfer live together. IEEE Commun. Lett..

[50] Zhao Y., Hu J., Diao Y., Yu Q., Yang K. (2018). Modelling and performance analysis of wireless LAN enabled by RF energy transfer. IEEE Trans. Commun..

[51] Khairy S., Han M., Cai L.X., Cheng Y. (2019). Sustainable wireless IoT networks with RF energy charging over Wi-Fi (CoWiFi). IEEE Internet Things J..

